# Supplementary material for: Cis-regulatory hubs: a new 3D model of complex disease genetics with an application to schizophrenia
Source: Life Sci Alliance. 2022 Jan 27;5(5):e202101156. doi: 10.26508/lsa.202101156 (PMC8807870; doi:10.26508/lsa.202101156)
Supplement: Supplementary file 1 [file LSA-2021-01156_Supplemental_Data_1.docx]

**Supplementary**
**Methods**
**3D Features**
**A/B Compartments**: A/B compartments were defined at a 500 kb resolution of the contact
matrix (using a 100 kb resolution had little impact on the results). The first principal
component (PC) of a suitably normalized Hi-C contact matrix over a chromosome arm
captures the plaid pattern of A/B compartments (Lieberman-Aiden et al., 2009). As GC
content is higher in A compartments than in B compartments, the correlation of the first PC
with GC content was used to orient the first PC so that positive values correspond to the A
compartments and negative values to B compartments (Imakaev et al., 2012). The
transformation applied to the ratio observed/expected (O/E) contact matrices was selected
that 1) maximized the number of autosomal chromosome arms where the first PC had the
highest correlation with GC content over the first three PCs and 2) had the highest correlation
of the first PC with GC content in these chromosome arms. The transformation was selected
among the following three: O/E - 1 with clipping of values below percentile 1 and above
percentile 99, log (O/E) and log (O/E) with clipping of values below percentile 1 and above
percentile 99. The last transformation was selected based on the above criteria, with 30/40
autosomal chromosome arms where the first PC correlated the most strongly with GC content
and correlations between 0.38 and 0.87 within these chromosome arms. The A/B
compartment for the remaining 10 chromosome arms (6q, 8q, 9p, 10q, 12p, 18p, 18q, 19q, 20p
and 21p) were set to missing.
**TADs calling**: TADs were called using the directionality index (Dixon et al., 2012), insulation
score (Crane et al., 2015) or with Arrowhead algorithm from Juicer software (Rao et al., 2014;
Durand et al., 2016)
*Directionality Index* (DI) was computed as presented by Dixon et al., 2012 at 10 Kb resolution.
Briefly, for each 10 Kb bins the number of upstream and downstream contacts were
calculated. A bias toward upstream regions at the end of a TAD was expected and conversely,
a bias toward downstream regions, at the beginning of a TAD was expected. As mentioned by
Gorkin et al., 2019, the original approach to computing the DI using a 200 Kb window size
was applied to capture more local features. DI values for each 10Kb bins were used to build a
Hidden Markov Model and predict upstream bias, downstream bias, and no bias states,
respectively. Regions switching from upstream bias to downstream bias were called
topological boundaries.
*Insulation Score* (INS) was computed as presented by Crane et al., 2015. Simply, for each 10
Kb bin, the average number of contacts in 400Kb windows upstream and downstream on O/E
matrices was computed. A local minimum for INS at TADs borders was expected. INS was
normalized at the chromosome level to take account of differences between chromosomes.
Then INS was scaled between 0 and 1, where 0 is complete insulation and 1 is no insulation
respectively.
*Arrowhead* TADs were annotated using Arrowhead (Rao et al., 2014; Durand et al., 2016) at 10
Kb resolution.
**Frequently Interacting Regions** (FIREs) (Schmitt et al., 2016) was computed with
FIREcaller R package (Crowley et al., 2021) at 10Kb resolution, with minor adjustments to fit
our data format.

**Functional Enrichment analysis**
Genes inside CRHs were used for GO enrichment analysis at the CRH or gene level. To do so,
at the CRH level the clusterProfiler R package (Wang et al., 2012) was used with the
compareCluster function to perform over-representation tests.

**Peak calling**
Peak calling for ChIP-Seq data was performed with MACS2 (Zhang et al., 2008) software through
the following command:
macs2 callpeak \
-t bamfile \
-n alias \
-f BAM \
-g hs \
-p .1 \
--call-summits \
--outdir outputdirectory

**Genome Build**
All coordinates in the human genome are reported using build hg19.

**Control Methods**
As mentioned in the main analysis, results were controlled using two other methods to
determine distal elements and CRHs. In doing so, a large spectrum of regulatory processes was
captured. Thus, alternative CRHs were defined through the Rao and DNAse methods, described
as follows.
**Rao**: Since it has been shown that significant 3D peaks are enriched in enhancers, all distal
elements were defined by 3D significant peaks linking promoters (Rao et al., 2014).
Briefly, as shown by Rao et al., 2014, a significant 3D peak is defined by comparing the
number of contacts in this 3D peak relative to four neighborhood regions: horizontal,
vertical, lower-left and donut, respectively.
**DNAse-based**: Since it has been shown that non-coding regions are open chromatin
regions, DNAse peaks were added as an additional biological layer to the 3D peaks from
the Rao method. In this method, distal elements are all DNAse peaks on 10Kb 3D peaks
in 3D contact with a promoter. Due to the methodology, several DNAse peaks can be
observed on the same 10Kb peak. Thus, each peak was considered as an individual distal
element.
Also mentioned in the main method section, we defined candidate CRHs in order to
assess comparison for enrichment analysis. For the Rao method, we considered all
significant 3D peaks in no contact with a promoter as candidate elements. Based on the same
rationale, DNAse candidate elements are all DNAse peaks in no 3D contact with a promoter.

**
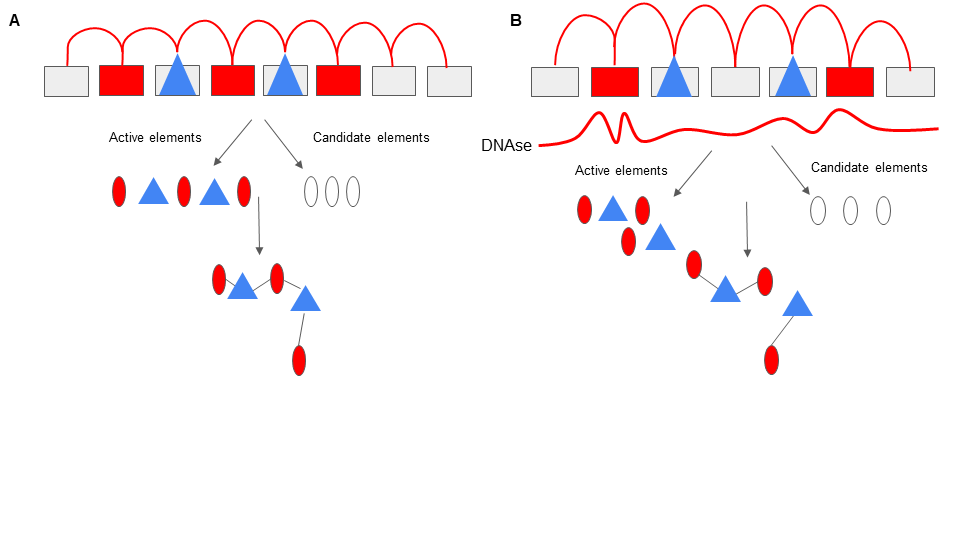
**

**Figure Supplemental Methods 1**

**(A)** Rao-Based method methodology and CRH building. **(B)** DNAse-based method methodology and CRH building

Data Information: Promoters are represented by blue triangles, distal regulatory elements by red circles and, candidate elements by white circles.

**HiC Mapping, Filtering, and Normalization for post-mortem brains**

Raw Hi-C sequence fastq files for post-mortem dopaminergic neuronal nuclei (NeuN+/Nurr1+) and the general neuronal (NeuN+) populations were obtained from the PsychENCODE Synapse platform. We referred in supplementary figures to Dopa_1 and Dopa_2 for post-mortem dopaminergic neuronal nuclei samples and Neu for the general neuronal sample, respectively. They were mapped to the human genome sequence (hg19) in 10 kb bins using distiller (<https://github.com/mirnylab/distiller-nf>). Genome-wide iterative correction (i.e., KR normalization) was performed using cooler (<https://github.com/mirnylab/cooler>). We used the hicConvertFormat tool from the HiCExplorer package ([https://hicexplorer.readthedocs.io](https://hicexplorer.readthedocs.io/)) to convert .cool files into ginteractions files, which after preprocessing were read by the Juicer toolbox and converted to .hic files.

**Acknowledgments**
The PsychEncode project is supported by: U01MH103392, U01MH103365, U01MH103346,
U01MH103340, U01MH103339, R21MH109956, R21MH105881, R21MH105853,
R21MH103877, R21MH102791, R01MH111721, R01MH110928, R01MH110927,
R01MH110926, R01MH110921, R01MH110920, R01MH110905, R01MH109715,
R01MH109677, R01MH105898, R01MH105898, R01MH094714, P50MH106934,
U01MH116488, U01MH116487, U01MH116492, U01MH116489, U01MH116438,
U01MH116441, U01MH116442, R01MH114911, R01MH114899, R01MH114901,
R01MH117293, R01MH117291, R01MH117292 awarded to: Schahram Akbarian (Icahn
School of Medicine at Mount Sinai), Gregory Crawford (Duke University), Stella Dracheva
(Icahn School of Medicine at Mount Sinai), Peggy Farnham (University of Southern
California), Mark Gerstein (Yale University), Daniel Geschwind (University of California, Los
Angeles), Fernando Goes (Johns Hopkins University), Thomas M. Hyde (Lieber Institute for
Brain Development), Andrew Jaffe (Lieber Institute for Brain Development), James A.
Knowles (University of Southern California), Chunyu Liu (SUNY Upstate Medical University),
Dalila Pinto (Icahn School of Medicine at Mount Sinai), Panos Roussos (Icahn School of
Medicine at Mount Sinai), Stephan Sanders (University of California, San Francisco), Nenad
Sestan (Yale University), Pamela Sklar (Icahn School of Medicine at Mount Sinai), Matthew
State (University of California, San Francisco), Patrick Sullivan (University of North
Carolina), Flora Vaccarino (Yale University), Daniel Weinberger (Lieber Institute for Brain
Development), Sherman Weissman (Yale University), Kevin White (University of Chicago),
Jeremy Willsey (University of California, San Francisco), and Peter Zandi (Johns Hopkins
University).

**References**
Crane, E., Bian, Q., McCord, R. P., Lajoie, B. R., Wheeler, B. S., Ralston, E. J., . . . Meyer, B. J.
(2015, 7). Condensin-driven remodelling of X chromosome topology during dosage
compensation. *Nature, 523*, 240–244. doi:10.1038/nature14450
Crowley, C., Yang, Y., Qiu, Y., Hu, B., Abnousi, A., Lipiński, J., . . . Li, Y. (2021). FIREcaller:
Detecting frequently interacting regions from Hi-C data. *Computational and Structural*
*Biotechnology Journal, 19*, 355–362. doi:10.1016/j.csbj.2020.12.026
Csardi, Gabor & Nepusz, Tamas. (2005). The Igraph Software Package for Complex
Network Research. InterJournal. Complex Systems. 1695.
Dixon, J. R., Selvaraj, S., Yue, F., Kim, A., Li, Y., Shen, Y., . . . Ren, B. (2012). Topological
domains in mammalian genomes identified by analysis of chromatin interactions.
*Nature, 485*, 376–380. doi:10.1038/nature11082
Imakaev, M., Fudenberg, G., McCord, R. P., Naumova, N., Goloborodko, A., Lajoie, B. R., . . .
Mirny, L. A. (2012, 10). Iterative correction of Hi-C data reveals hallmarks of
chromosome organization. *Nature Methods, 9*, 999–1003. doi:10.1038/nmeth.2148
Lieberman-aiden, E., Berkum, N. L., Williams, L., Imakaev, M., Ragoczy, T., Telling, A., . . .
Mirny, L. A. (2009). of the Human Genome. *33292*, 289–294.
Rao, S. S., Huntley, M. H., Durand, N. C., Stamenova, E. K., Bochkov, I. D., Robinson, J. T., . . .
Aiden, E. L. (2014). A 3D map of the human genome at kilobase resolution reveals
principles of chromatin looping. *Cell, 159*, 1665–1680. doi:10.1016/j.cell.2014.11.021
Schmitt, A. D., Hu, M., Jung, I., Xu, Z., Qiu, Y., Tan, C. L., . . . Ren, B. (2016). A Compendium of
Chromatin Contact Maps Reveals Spatially Active Regions in the Human Genome. *Cell*
*Reports, 17*, 2042–2059. doi:10.1016/j.celrep.2016.10.061
Yu G, Wang L, Han Y, He Q (2012). “clusterProfiler: an R package for
comparing biological themes among gene clusters.” OMICS: A Journal of Integrative
Biology, 16(5), 284-287. doi:
Zhang, Y., Liu, T., Meyer, C. A., Eeckhoute, J., Johnson, D. S., Bernstein, B. E., . . . Shirley, X. S.
(2008). Model-based analysis of ChIP-Seq (MACS). Genome Biology, 9. doi:10.1186/gb-
2008-9-9-r137
